# Behçet's Disease (Adamantiades-Behçet's Disease)

**DOI:** 10.1155/2011/681956

**Published:** 2010-11-01

**Authors:** Fumio Kaneko, Ari Togashi, Sanae Saito, Hideo Sakuma, Noritaka Oyama, Koichiro Nakamura, Kenji Yokota, Keiji Oguma

**Affiliations:** ^1^Institute of Dermato-Immunology and Allergy, Southern TOHOKU Research Institute for Neuroscience, 7-115 Yatsuyamada, Koriyama, Fukushima 963-8563, Japan; ^2^Pathology Division, Southern TOHOKU Research Institute for Neuroscience, 7-115 Yatsuyamada, Koriyama 963-8563, Japan; ^3^Departments of Dermatology, School of Medicine, Fukushima Medical University, Hikarigaoka-1, Fukushima 960-1295, Japan; ^4^Saitama Medical University, 38 Hongo, Moroyama, Iruma-gun, Saitama 350-0495, Japan; ^5^Department of Bacteriology, Graduate School of Medicine and Dentistry, Medical School, Okayama University, 5-1, Shikata-cho-2, Okayama 700-8558, Japan

## Abstract

Adamantiades-Behçet's disease (ABD) is characterized by starting with oral aphthous ulceration and developing of the systemic involvements. The pathogenesis of ABD is closely correlated with the genetic factors and the triggering factors which acquire delayed-type hypersensitivity reaction against oral *streptococci* mediated by IL-12 cytokine family. HLA-B51 is associated in more than 60% of the patients and its restricted CD8+ T cell response is clearly correlated with the target tissues. *Bes-1* gene encoded partial *S. sanguinis* genome which is highly homologous with retinal protein, and 65 kD heat shock protein (Hsp-65) released from streptococci is playing an important role with human Hsp-60 in the pathogenesis of ABD. Although Hsp-65/60 has homologies with the respective T cell epitope, it stimulates peripheral blood mononuclear cells (PBMCs) from ABD patients. On the other hand, some peptides of Hsp-65 were found to reduce IL-8 and IL-12 production from PBMCs of ABD patients in active stage.

## 1. Introduction

Behçet's disease [1] (Adamantiades-Behçet's disease [2–4]) (ABD) is a chronic and multisystematic inflammatory disorder characterized by starting with oral ulceration and develops the recurrent involvement of mucocutaneous (oral and genital ulceration, acne-like eruption, erythema nodosum- (EN-) like eruption, etc.), ocular, vascular, digestive, and/or nervous system organs. Although the actual etiology is still unclear, ABD symptoms are considered to be based on the correlation between the genetic intrinsic factors and the triggering extrinsic factors, because more than 60% of ABD patients are associated with HLA-B51 [2–4]. As one of the triggering extrinsic factors, the oral unhygienic condition may be suspected, because periodontitis, decayed teeth, chronic tonsillitis, and so forth are frequently noted in the oral cavity of ABD patients [5–7]. The proportion of *Streptococcus sanguinis* (*S*.  *s* *a* *n* *g* *u* *i* *n* *i* *s*) and *S. mitis* was pointed to significantly increase in the oral bacteria flora of ABD patients in our country [8–10]. *S. sanguinis* was previously recognized as *species *of the *genus* Streptococcus named “*S. sanguis*” and the strain isolated from ABD patients was different from reference ATCC strains in DNA homology and sugar constituents, which indicate uncommon serotype KTH-1 (so-called BD113-20 strain) [8–10]. Regarding the increasing of the microorganism in the oral cavity of ABD patients, it may be different from ethnic population, because *S. mutans* is reported to be increased in Turkey [7]. Most of the patients including recurrent aphthosis (RA) tend to acquire delayed type hypersensitivity (DTH) against *streptococci* [5, 6, 11–13]. The serum-antibody titers against *streptococci* were also elevated in ABD patients [5–7]. The 65 kDa of a heat shock protein (Hsp-65) related to *S. sanguinis* can be detected along with counterpart human Hsp-60 which might reactively appear in the sera and lesions of ABD patients [14, 15]. The lesions are histologically considered to be DTH reaction with perivascular mononuclear cell infiltration, but neutrophils are also infiltrated in the early stage, as seen in EN-like eruption [12, 16, 17]. Especially, the mucous epithelial cells of the oral ulceration, which express streptococcal antigen and adhering molecules, are interstitially infiltrated by mononuclear cells and neutrophils [12, 16]. Generally, interleukin (IL)-12 produced by the infiltrated mononuclear cells, which might be antigen presenting cells (APCs), is thought to induce naive T (Th0) cells to T-helper type-1 (Th-1) cells [18] in the correlation with DTH reaction. 

Hence, in this paper, we would like to focus on the role of immune reactions against oral *streptococci* mediated by IL-12 cytokine family in the pathogenesis of ABD.

## 2. Oral Streptococci and Systemic Symptoms in ABD Patients

In the oral cavity of ABD patients,* streptococci* are significantly increased [7–10] and ABD and RA patients have hypersensitivity against them, as above described [5, 6, 11–13]. Then, we tried to prick with their self-saliva (salivary prick: S-prick) on the forearm skin of ABD patients using a “prick-lansetter” with a tiny stick (OY Algol Ab Espoo/Esbo Puh90-50991, Sweden) to avoid so-called “pathergy reaction”, because *streptococci* are naturally contained in the saliva [19]. The pathergy test has been considered as a mysterious characteristic and diagnostic measure for ABD patients for long time, and the reactive phenomena might be suggested as one of autoimmune disorders. However, the reaction is seen in 30%–40% of the patients even though the thick syringe-needle around “20 G” is used and is not always diagnostic for ABD patients in our country [20]. The histology of the reaction suggests DTH reaction with vascular changes infiltrated by mononuclear cells as seen in EN-like eruption of ABD patients [19]. 

**Figure 1:**
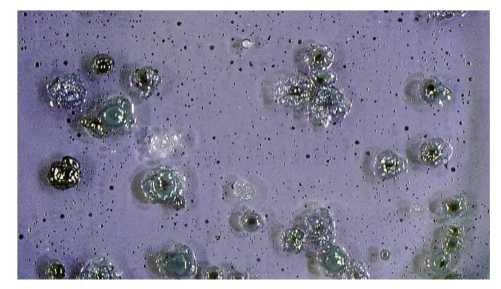
Streptococcal colonies appeared from saliva of an ABD patient in MS agar dish. According to the instruction of the MS agar (Difco Lab.), mainly 3 kinds of bacterial colonies are differentiated as blue crystal (probably *S. mitis,* etc.), gum-drop (*S. salivarius,* etc.), and dip (micrococcus species, etc.) shapes at 3–5 day culture.

**Figure 2:**
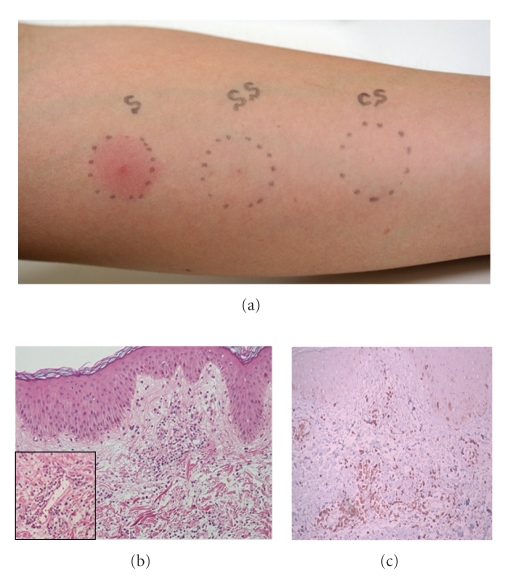
(a) 33-year-old female ABD patient showed erythema reaction exhibiting more than 10 mm in diameter by self-saliva (salivary prick: S-prick) 48 hours after prick. A tinny spot by sterilized self-saliva (sterilized salivary prick: SS-prick) and control saline (control saline prick: CS-prick) were observed. (b) Histology of the skin reaction by S-prick from 27-year-old male ABD patient. Edema in the upper dermis and many mononuculear cell infiltration around the dilated vessels in the middle dermis were found (HE, 100x). The magnified view of the vascular change with infiltrate (200x). (c) Immunochemical histology of the skin reaction by S-prick. (avidin-biotin complex peroxydase method: ABC method, 200x). Infiltration of CD3+/CD4+ cells is mainly found around vessels and the proportion of infiltrate was CD3+/4+ > CD68+ > CD8+ cells. No CD20+ and CD56+ cells were found.

The oral *streptococci* can be generally observed as main 3 kinds of streptococcal colonies appearing at 3–5 day incubation of the saliva in MS (Mitis-Salivarius) agar with 1% tellunite solution dish which *streptococci* are selectively grown (Difco Lab., Detroit, USA) (Figure 1). Forty-eight hours after S-prick, the DTH reaction appeared at the prick site in more than 70% of probability in ABD patients (Figure 2(a)) [19]. Since the skin reaction did not appear by the sterilized saliva (SS-prick) using the syringe filter with 0.2 *μ*m (Nalgene, Nunc International Co., USA), the S-prick reaction is considered to be due to oral *streptococci*. The histology of the biopsy specimen from the S-prick reaction shows edema in the upper dermis and mononuclear cell infiltration around and in the walls of the vessels in the dermis (Figure 2(b)). The infiltrated mononuclear cells were mainly composed of CD3+/CD4+ cells > CD68+ cells (Figure 2(c)) >CD8+ cells and no CD20+ and CD56+ cells were found, suggesting DTH reaction. This histologic findings seem to be similar to that of the pathergy reaction exhibiting vascular changes [21] and also similar to that of EN-like eruption appeared on the lower leg of ABD patients [12, 16, 17]. Generally, in EN-like eruption, mononuclear cells were infiltrated around vessels in the middle dermis of the biopsy specimen which was taken at 5th day after the appearance and the infiltrated cells were mainly composed of CD3+/CD4+ cells >CD8+ cells > CD68+ cells. These results provide evidence that S-prick is highly diagnostic for ABD patients and also suggest that the pathergy reaction might be due to cutaneous bacteria having cross immunity to oral *streptococci*.

## 3. HLA Genotyping and Streptococcal Hypersensitivity

HLA-B51 is supposed to be a highly associated genetic marker of ABD patients from many different ethnic groups including European, Mediterranean, and Asian people [2–4, 22, 23]. ABD has several unique epidemiologic features distributed from Southern Europe to Japan along “the old Silk Route” in accordance with HLA distribution [3, 4, 23]. It is of interest that HLA-B51-transgenic mice show enhanced neutrophil function as seen in ABD patients because HLA-B51 gene presents at the endogenous peptides of CD8+ T cells (regulatory T cells: T reg cells), although these mice did not express the symptoms of ABD [24]. Regarding the extrinsic factor, Hsp-65/60 related to the infection by *streptococci* and the affected tissues is detectable in the oral mucosal ulceration and other lesions of ABD patients [14, 15]. 

Generally, APCs, which produce IL-12 in correlation with DTH reaction, are thought to be activated in the peripheral blood mononuclear cells (PBMCs) of ABD patients with HLA-B51 in active disease stage, as indicated by Yasuoka et al. [25]. On the other hand, we have obtained interesting results that PBMCs from ABD patients in negative HLA-B51, by stimulation with *S. sanguinis* antigen, exhibited more significant expression of IL-12p40 (IL-12B) mRNA and increased protein level of IL-12p70 (70 kDa composed of p35 and p40 subunits) than those of the patients with HLA-B51. IL-12B promoter genotype of ABD patients was also analyzed by polymerase chain reaction- (PCR-) based restriction enzyme digestion. The results suggested the frequency of the insertion heterozygosity was significantly higher in ABD patients without HLA-B51, which indicates the presence of IL-12B gene polymorphism in the promoter region. The reaction to *streptococci* toward Th1-immunity mediated by IL-12 in ABD patients seemed to be different from HLA background [26].

The oral health is impaired in ABD patients, which seems to be associated with the disease severity [5, 7, 9, 28]. The uncommon serotype (KTH-1) *S. sanguinis* is significantly increased in the oral bacterial flora of ABD patients compared to healthy individuals (HIs) [8, 9], and the serum antibodies also showed cross reactivity with the some peptides of Hsp-65, as previously described [29]. It has been demonstrated that the symptoms mimicking ABD appeared in germ-free mice when *S. sanguinis* from ABD patients was inoculated into their oral tissues. The immunization with *S. sanguinis* through the oral membrane route is suggested to elicit ABD-like symptoms in the animal model as seen in ABD patients who carry *S. sanguinis* in the oral cavity [30]. Interestingly, the presence of *Bes-1* DNA encoded *S. sanguinis* gene was found in various mucocutaneous lesions including oral and genital ulcerations and EN-like lesions and, besides, PCR in situ hybridization revealed that *Bes-1* DNA was expressed in the cytoplasm of the infiltrated mononuclear cells adhering the vascular walls in the lesions [31]. The peptide of Hsp-60 (336–351) was found to be correlated with the peptide of *Bes-1* (229–243) and the peptide of *Bes-1* (337–385) can stimulate and produce IFN-*γ* and IL-12 from PBMCs of ABD patients [29]. 

## 4. Role of Hsps-65/60 in Oral Lesions

Hsps, which essentially scavenge denatured intracellular proteins, are supposed to be induced by microorganisms and mammalian tissues under a variety of stressful condition [33], and they may be involved in the pathogenesis of some autoimmune diseases [34]. In ABD patients, the serum levels of IgA antibodies to mycobacterial Hsp-65, which cross reacts with selected strains of *S. sanguinis*, are increased significantly [35, 36]. APCs taken Hsps are thought to release IL-12 and stimulate T cells and the Hsp-65/60 expressed monocytes, which are detectable in various ABD lesions, lead T cells to undergo apoptosis after IFN-*γ* production [32, 35–43]. Four peptides of Hsp-65 (111–125, 154–172, 219–233, and 311–326) derived from *S. sanguinis* were recognized as immune binding agents for T and B cell responses and the peptides showed 50%–80% homology to the counterpart human Hsp-60 (Table 1) [41–43]. These Hsps presented by APCs can directly stimulate *α* *β*T and *γ* *δ*T cells which play important roles in the oral mucosal immunity as the first defense against microorganisms. It is thought that V*γ*9*δ*2+ T cells, a major subset of *γ* *δ*T cells in PBMCs, recognize the antigens produced by bacteria and that innate and adaptive immune responses are influenced by secreting IFN-*γ* from these cells [44]. These *γ* *δ*T cells seemed to be elevated in the blood flow and mucocutaneous lesions of ABD patients and these activated APCs and *γ* *δ*T cells might activate *α* *β*T cells by their secretion of proinflammatory cytokines in the lesions [16, 44].

## 5. Toll-Like Receptor (TLR) in the Innate Immunity

Regarding the recognition system for the microorganism antigens in humans, 10 numbers of Toll-like receptor (TLR) family are supposed to act as innate immune receptors by binding of particular structures present on bacteria, viruses, fungi, and so forth [45]. Although the expression of TLRs has been detected in various human tissues with varying levels, the organs involved in immune response and exposed to environment were found to have significantly higher TLR expression [46]. TLR-3 (ds RNA) and TLR-6 (mycoplasma, staphylococci, etc.) are also reported to be enhanced in expression on neutrophils and monocytes of ABD patients, when stimulated by Hsp-60 and *S. sanguinis* antigen [47]. APCs expressing streptococcal antigen by which took Hsps and *Bes-1* gene through the TLRs in the oral cavity are supposed to cause the immunological reaction as the lesions of ABD patients when they were curried peripherally and adhered to the impaired endothelium of the vessels.

## 6. Therapeutic Device

As the pathogenesis of ABD was described as above, the DTH reaction against oral streptococcal antigens, which might be gained by APCs through the innate immune mechanism in the oral cavity, takes place in the various lesions of ABD patients. Generally, ABD patients have been treated with the antisymptomatic drugs as followings; immunosupressants such as nonsteroid anti-inflammatory agents, steroids, colchicine, cyclosporine A, and so forth. However, recently antitumor necrosis factor (TNF)-*α* antibody has been newly added as a therapeutic tool for ABD patients with severe uveitis [48]. On the other hand, the antibiotics to the oral *streptococci* were also effective, and especially, minocycline reduced not only the growth of *streptococci*, but also cytokine production from activated T cells [6]. Other studies also reported that combination therapy, colchicines, and benzathine penicillin was effective to suppress ABD symptoms compared to colchicine monotherapy [49, 50]. 

It is of interest that the peptide of Hsp-60 (336–351) linked to recombinant cholera toxin B (rCTB) subunit reduced the uveitis induced by whole Hsp-60 in Lewis strain rats, although the peptide without adjuvant is reported to induce uveitis [42, 43]. A therapeutic trial with the selective peptide conjugated with rCTB was done by oral administration for ABD patients with severe uveitis. In those patients who were controlled from the symptoms, a lack of the peptide-specific CD4+ T cell population, decrease in expression of Th1 type cells, and reduction of cytokines from T cells were found [43]. Hsps might trigger both innate and adaptive immune mechanisms in ABD, but on the other hand, the therapeutic approaches involving Hsp immunomodulation may be available as “oral toleration” for ABD patients with advanced uveitis using the peptide of Hsp (336–351) [43].

**Figure 3:**
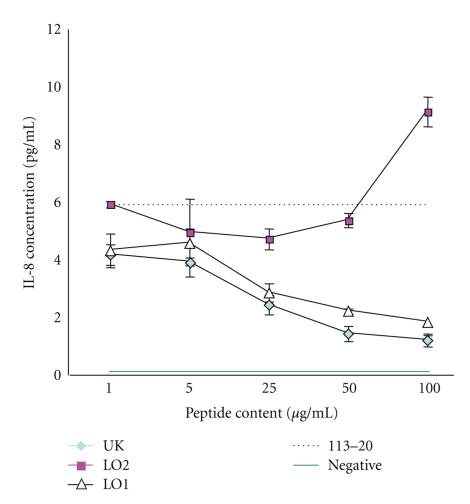
IL-8 production from MONO cells activated by *S. sanguinis* antigen was suppressed by LO1 and UK in a dose-dependent manner.

Then, we analyzed Hsp-65 derived from *S. sanguinis* to find the homologous peptides to T cell epitope of ABD patients and also to find how the peptides reduce the production of cytokines from PBMCs of ABD patients in active disease stage. The 4 peptides of Hsp-65 were shown to significantly stimulate and undergo CD4+ and CD8+ T cell apoptosis in PBMCs from ABD patients. Hsp-60 also seemed to stimulate them similarly, because the peptide of Hsp-60 (336–351) was identified to be highly homologous to T cell epitope [14, 35–39]. The peptides LO1 (249–264), IIIa (365–384), IIIb (395–413), LO2 (480–499), LO3 (504–518), and UK (311–326) corresponding to the human Hsp60 (336–351) (Table 1) were applied for activated T cells of ABD patients in vitro to lead immunotoleration. PBMCs from 7 active ABD patients and 5 HIs were incubated with and without these peptides and 7 days after incubation mainly IL-8 and IL-12 were measured and compared with those from PBMCs of active ABD patients incubated without the peptides as controls. Although IL-12 and IL-8 were naturally produced from PBMCs of active ABD patients, the significant reduction of inflammatory cytokines was found by the 5 peptides, LO1, LO2, LO3, IIIb, and UK. To understand the suppressive mechanisms of the cytokine production in PBMCs from active ABD patients, we tried to find the binding sites of the peptides on monocytes by cDNA chips (Gene Chip; Human Genome) using NOMO-1 cells (human macrophage cell line) which were activated by *S. sanguinis* antigen. The NOMO-1 cells incubated with LO1 were suppressed to produce IL-8 (Figure 3). CD58 molecule and/or FK506-binding protein were highly expressed on the cell surface of the NOMO-1 cells by LO1 [27, 32]. It is considered that activated T cells of ABD patients might be led to apopthosis by binding of LO1 on the receptors of the cells. 

## 7. Conclusions

**Figure 4:**
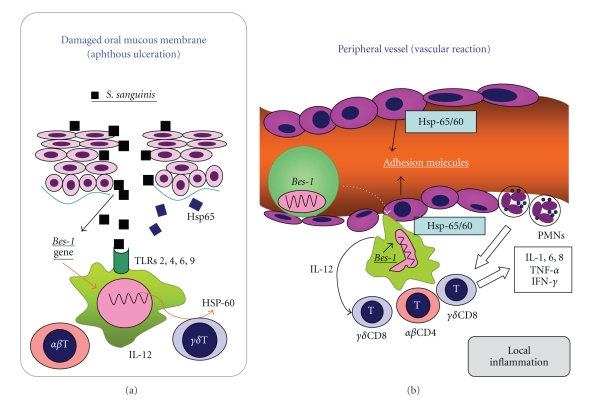
(a) and (b) Hypothesis of oral immune reaction developing the systemic manifestation in ABD. The local vascular reaction may be caused by the antigen presenting cells immunized by *S. sanguinis* in the oral cavity [32].

 Although the etiology of ABD is still obscure, the clinical manuscripts generally start with oral aphthous ulceration and develop systemic involvement. The close correlation between the genetic internal and triggering external factors is thought to be present in the pathogenesis of ABD. Most of ABD patients tend to acquire hypersensitivity against *streptococci* in the oral cavity and express DTH reaction mediated by IL-12 in the various lesions. It might be explained that when the APCs taken streptococcal agents were locally curried and adhered to the damaged vessel walls, the DTH response developed to the various clinical symptoms (Figures 4(a) and 4(b)). The cutaneous DTH reaction by S-prick is suggested to show the mechanism of the regional symptoms and also the S-prick is thought to be highly diagnostic for ABD patients rather than the pathergy test. Hsp-65/60 seems to play an important role in the pathogenesis of ABD. The probability of a new therapy for ABD patients was discussed as the immune tolerance utilizing the peptides of Hsp-65/60. 

## Figures and Tables

**Table 1:** Synthetic peptides of Hsp-65 derived from *S. sanguinis*. MW: molecular weight (daltons) (Oguma et al., 2008) [27].

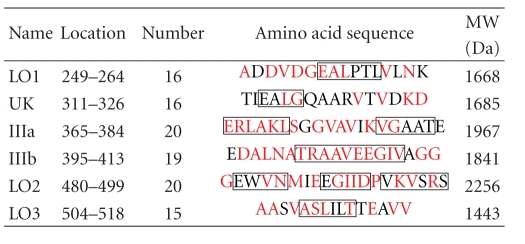

## Abbreviations

ABC: Avidin-biotin complex
ABD: Adamantiades-Behçet's disease
APCs: Antigen presenting cells
DTH: Delayed type hypersensitivity
EN: Erythema nodosum
HIs: Healthy individuals
Hsp: Heat shock protein
IL: Interleukin
MS: Mitis-Salivarius
PBMCs: Peripheral blood mononuclear cells
PCR: Polymerase chain reaction
RA: Recurrent aphthosis
S-prick: Prick with self-saliva
SS-prick: Prick with sterilized self-saliva
TLR: Toll-like receptor.
